# Regional Disparity of Medical Resources and Its Effect on Mortality Rates in China

**DOI:** 10.3389/fpubh.2020.00008

**Published:** 2020-02-04

**Authors:** Kuang-Cheng Chai, Ying-Bin Zhang, Ke-Chiun Chang

**Affiliations:** ^1^Business School, Guilin University of Electronic Technology, Guilin, China; ^2^School of Economic and Management, Wuhan University, Wuhan, China

**Keywords:** mortality rate, medical resources, regional disparity, unemployment, GDP

## Abstract

**Objectives:** The purpose of this study was two-fold. First, to empirically study the effects that medical resources (i.e., hospital, doctors, beds) have on the mortality rate in China. Second, to divide China into east, middle, and west regions, and empirically study the regional disparity of medical resources and its effect on mortality rates in China.

**Methodology and Data:** This study utilized a panel data regression model to explore the effect medical resources have on the age-standardized mortality rate in China. The data came from the 2003–2017 China Statistical Yearbook compiled by the National Bureau of Statistics of China.

**Results:** Nationwide, hospitals, doctors, and beds had a significant negative correlation with the mortality rate. In the western region, hospitals, beds, and doctors had a significant negative correlation with the mortality rate. In China's middle and eastern regions, hospitals, beds, and doctors had no significant effect on the mortality rate. In China, increased hospitals, doctors, and beds significantly reduced the mortality rate. The distribution of medical resources in eastern, middle, and western China was unequal. More hospitals, beds, and doctors in the less developed western regions can more effectively alleviate the local mortality rate. In the middle and east regions, hospitals, beds, and doctors had no significant impact on the local mortality rate.

**Conclusion:** First, China's overall medical resources are still inadequate and improving medical resources throughout the country could reduce the mortality rate. Second, due to the imbalanced distribution of medical resources in China, the Chinese government should implement more supportive policies for medical resources in the western region. At the same time, we should also actively develop the western region by improving local per capita GDP and reducing unemployment, so as to fundamentally reduce the local mortality rate.

## Introduction

Mortality rate is a common indicator for evaluating health status, and medical access is the most important factor in studies on mortality or health (1). Improving health, such as lower mortality rates, has had a major social benefit. From the perspective of patients receiving medical treatment, there are two main factors affecting the medical impact on patients. The first is the regional accessibility of medical organizations, and the second is the amount of medical resources invested by medical organizations. The common indicators to evaluate these two factors are the ratio of medical human resources to the number of medical facilities, and the number of doctors, hospitals, and beds per capita. In this context, the regional distribution of medical resources has become an important social issue. Therefore, it is necessary to systematically and quantitatively study the relationship between the regional distribution of medical resources and mortality in China.

Although China is developing rapidly, the uncoordinated development across different regions is a serious problem. Nationwide, medical resources are mainly distributed to economically developed eastern provinces and densely populated middle provinces. Conversely, the economic development of the western province is backward, where the area is vast and the population is relatively sparse. This results in a lack of medical resources in the western region and this lack of medical resources has a negative impact on local health. Therefore, regional economic conditions is a factor in the unequal distribution of medical resources across regions. In areas with better economic conditions, medical resources are more concentrated and there are more doctors, hospitals, and beds. On the other hand, the concentration of such resources is relatively low or non-existent in regions with weaker economies. Therefore, it is likely that such differences in concentrations or unequal distribution will naturally lead to differences in medical outcomes, with an impact on local mortality. In this context, the regional characteristics of medical resources are undoubtedly related to local mortality rates and can be said to have very important implications.

## Literature Review

Most of the research on mortality has focused on specific diseases, natural climates, and crises (2–4). Some studies of mortality focused on the categories of death on the basis of the analysis of the existing materials of the cause of death (5–8). However, studies on the impact of socio-economic factors and regional characteristics on mortality have been emerging (9–11). These studies focused on regional differences, or changes in health levels, and found significant associations between regional differences in health levels, particularly in regional mortality. Many studies have shown that socio-economic factors, rather than natural environment, play an important role in contributing to regional differences in mortality (6, 8, 12–16). Their findings empirically proved the influence of medical resource on individual health level. Soares (17) in his systematic in-depth discussion of mortality-reducing factors in developing countries, mentioned that public health infrastructure, vaccination, target projects, and the dissemination of knowledge play a very important role in distribution, but these all depend on local medical resources. Anoushiravani et al. (18) and Schidlow et al. (19) study the impact of medical resources on mortality. They analyzed the impact of medical resources (number of doctors, hospitals, beds) and used panel regression models to derive different indicators of health performance outcomes (life expectancy, mortality, years of potential life lost). Their findings suggest that increased medical resources actually do significantly reduce mortality, and thus have a positive impact on health levels. Studies have also examined the effects of macroeconomic variables, such as regional unemployment and income levels, on health and mortality levels (8, 9, 20).

Access to medical resources is also closely related to significant regional differences in health levels or mortality. This is due to the positive correlation between areas with high medical resources supply and high income pointed out by Lee and Kim (21). Even if regional differences in medical resources and their effects on other factors are controlled, access to physical and human medical resources will be unbalanced. Cutler et al. (22) claim that medical resources in some regions were overflowed and wasted, while some were insufficient. This is bound to have an impact on the health of local populations in areas with inadequate medical resources. Preston (23) puts forward that in higher income areas, the increase of population investment in health can help local people improve their health level. Therefore, promoting the mobility of the rich to the economically backward areas can improve the level of medical resources in the backward areas. James and Cossman (24) finds that the impact of regional health conditions and socio-economic environment on regional mortality rate is greater than that of medical progress, and further suggests that the supply of medical resources in backward areas should be increased. Liu and Zhang (25) pointed out that regional medical resources are unbalanced from demographic and socioeconomic perspective. The unbalanced distribution of medical resources leads to unfair curative effect, which affects the overall health level of the whole nation. Grigoriev and Pechholdová (26) compared eastern and western medical resources in German, and point out that improved medical resources in poor areas can significantly reduce mortality.

This study focused on the geographical distribution of medical resources and its impact on the mortality rate of local residents from a national perspective. The objective is on the following three aspect: first, to compare the differences of medical resources, mortality rates, GDP, and unemployment rates in eastern, middle and western China through descriptive statistical comparison; second, to provide an empirical study on the overall impact of China's domestic medical resources on mortality; and third, to provide empirical research on the impact of medical resources on the local mortality rate in east, middle and west China and to present evidence that shows the difference in regional mortality rates is caused by an unequal distribution of medical resources in China. Lastly, this study concludes with policy implications.

## Hypotheses

Population mortality is influenced by many factors, which are not only determined by individual, but also impacted by social environment and natural environment (2–4, 8, 10, 11). As important indicator of social resources, medical resources include hospitals, doctors, and beds. According to Grossman (27) health of the human capital theory, health spending is the importance of a healthy capital input, the individual's environment is rich in medical resources will improve the accessibility of medical resources, reduce barriers to go to a doctor to better meet the demand of individual medical treatment which is beneficial to the health capital accumulation and mortality reduction. Aday and Andersen (28) mad the earliest medical accessibility measure applied to the utilization of health services research, they point out that the medical accessibility has potential level and practical level. Potential accessibility refers to the obstacles that the innate environment or acquired ability of the patient affects the access to medical services, while practical accessibility refers to the utilization of medical resources and subjective feelings of the patient in the process of receiving medical services. Due to the difficulty of subjective perception measurement, subsequent studies on actual accessibility mainly focus on the utilization of medical resources. From the perspective of the process of patients receiving medical treatment, there are two main factors influencing the medical effect of patients. The first is the amount of medical resources input by medical organizations, and the second is the geographical accessibility of medical organizations. Common indicators for evaluating the first factor are the ratio of medical human resources to the population of medical facilities, such as the number of doctors per capita, hospitals, beds, and so on. Common indicators to evaluate the second factor include transportation time, appointment waiting time, etc. Nearby medical resources can improve the medical accessibility of local residents, while remote medical resources will reduce the medical accessibility and increase the mortality rate (26, 29, 30). Adequate local medical resources can improve medical access for local residents and reduce local mortality.

The geographical accessibility of medical services is an important indicator that affects the mortality rate. Lower transportation time and treatment time will increase the probability of treatment for residents and reduce the mortality rate. Liu and Zhang (25) pointed out that regional medical resources are unbalanced from demographic and socioeconomic perspective. The unbalanced distribution of medical resources leads to unfair curative effect, which affects the overall health level of the whole nation. There are many factors impacting medical resources, among which the local economic level is a very important factor. Preston (23) puts forward that in higher income regions, the increase of population investment in health can help local people improve their health level. Poor regions struggle to improve health care because of lack of funds (31, 32).

With the rapid improvement of China's living standards, as well as economic development, the demand for medical resources is increasing day by day. At present, the allocation problem of social medical resources in China has changed from overall shortage to regional inequality. Admittedly, the overall medical resources in China still need to be improved, but the medical resources in west region are scarcer. Death rates are also relatively high in western region. The paper divide China as three part: eastern, middle and western region based on classification of China Statistical Yearbook. The GDP in eastern region is generally high and the economy is relatively developed; the GDP in middle region is generally medium and the economy is relatively developing; the GDP in western region is generally low and the economy is relatively backward. Scholars have empirically demonstrated that increased medical resources can significantly reduce local mortality (20, 23, 25, 31–33). There are still significant problems of uncoordinated regional development in China. Table 4 show that there is relatively scare medical resource in the west. Western residents are lack of medical access, which has impact on local residents' mortality rate. Relatively, residents in east and middle have better medical access. Liu and Zhang (25) pointed out that the unbalanced distribution of medical resources leads to unfair curative effect, which affects the overall health level of the whole nation. Grigoriev and Pechholdová (26) compared eastern and western medical resources in German, and point out that improved medical resources in poor areas can significantly reduce mortality. Therefore, increasing west region's medical resource could better improve medical access for local residents and better reduce mortality rate than in east and middle region in China. Based on China's current situation, this study proposes the following hypotheses:

*Hypothesis 1*: *Each medical resource will reduce mortality rate in China*.
*Hypothesis 2*: *The influence that medical resources have on the mortality rate is more significant in the west region than in the middle and east regions*.

## Materials and Methods

### Analysis

The data came from the 2003–2017 China Statistical Yearbook compiled by the National Bureau of Statistics of China. The changes in doctors, hospitals, beds, and death rates in each province were examined by looking at a statistical Yearbook of China. In order to analyze the validity of these factors, the fixed effect model was adopted. As mentioned in numerous studies of panel models, the fixed effect model considers the characteristics of a population not observed in the established panel data as a fixed parameter, and only assumes that a cross section depends on a cross section element or a population. The advantage of this method is that it solves the endogenous problem of the relationship between unobservable characteristics and mortality rate. In addition, this study considers that the impact of medical resources on mortality is affected by time, so it fixes the time effect. By controlling for socioeconomic variables in each region, more relevant outcomes can be estimated that better reflect the impact of each health resource on mortality.

These hypotheses are closely related to the quantitative analysis model in this study. Our objective was to investigate the impact of each medical resource on mortality (fixed effects model). According to the China Statistical Yearbook classifications, Chinese provinces were divided into east, middle and west groups for regression (fixed effect model).

### Specification

Variables in this study refer to Chang and Kim (33) and Chan et al. (34), and set medical resource per capita (i.e., doctors, hospitals, and beds) as independent variables. The per capita GDP, inflation of each province was set as a control variable to overcome the endogenous problem generated by socio-economic conditions. Birth rate and population density were set as control variable to overcome the endogenous problem generated by demographic conditions. Referring to Lee and Kim (21) the unemployment rate was set as a control variable to overcome the endogenous problem caused by the difference in the availability of medical resources across regions. These variables have been used in many previous studies as key variables that reflect the socio-economic characteristics of an area. Table 1 shows the variables and variable descriptions for this study. As mentioned earlier, the amount of all medical resources is standardized according to the population of each province. The following equation describes the basic structural model of analysis:

(1) $$\begin{array}{l} {Mortality_{it}=\omega_{i}+\gamma_{t}+\beta }_{1}{Medical\;Resource_{it}+\beta }_{2}Control_{it}\\ +\underset{m}{\sum }\lambda_{m}Province_{m}+\underset{n}{\sum }\varphi_{n}Year_{n}+\varepsilon_{it} \end{array}$$

Equation (1) represents the relationship between mortality and a medical resource. Medical resource represents hospital, bed, and doctor. The control represents GDP, unemployment, inflation, density, birth, *i* represent area, *t* represent time, ω_i_ represent the fixed effect of area, and γ_t_ represent the fixed effect of time trends.

**Table 1:** Construction of data.

**Variable**	**Description of variable**
Mortality	Death rate of residents*100 by province. Unit of value is percentage point
Hospital	Number of hospitals per residents by province. Unit of value is number/per person
Bed	Number of beds per residents by province. Unit of value is number/per person
Doctor	Number of doctors per residents by province. Unit of value is number/per person
Unemployment	Unemployment rate of residents by province. Unit of value is percentage point
GDP	Per Capita GDP of residents by province. Unit of value is 10,000 yuan
Inflation	The inflation by province. Unit of value is percentage point
Density	The population by province/area by province. Unit of value is number of person/10,000 square meter
Birth	Birth rate of residents by province. Unit of value is percentage point

## Results

### Descriptive Statistics and Correlation Matrix

The descriptive statistics on variables introduced to our model is shown in Table 2. In a sample of 31 provinces in China from 2003 to 2017, the national average mortality rate was 5.95 percentage points. In the east, it was 5.69, lower than the national average. Meanwhile, the middle and west regions were both higher than the national average at 6.09 and 6.07, respectively. The average residents of hospitals, doctors, and beds residents in China were 0.01, 0.01, 0.03; in the east region, 0.01, 0.02, and 0.04, respectively; in the middle region, 0.01, 0.02, and 0.04, respectively; and in the west region, 0.001, 0.002, and 0.01, respectively. The per capita GDP of the eastern provinces was higher than the national level, while the per capita GDPs of the middle and west regions were lower than the national level. Compared to national rates, unemployment rates were lower in the east and higher in the central and western regions. Descriptive statistics show that the middle and east regions are similar in terms of medical resources, while the west lags far behind them. GDP and unemployment rates in the middle and western regions lagged behind those in the east. Population density is higher in the east than in the middle and western regions.

**Table 2:** Descriptive statistics.

**Variable**	**Classification**	**Mean**	**SD**	**Min**.	**Max**.	**Observed**
Mortality	Overall	5.95	0.70	4.20	7.40	465
	East	5.69	0.69	4.21	7.40	150
	Middle	6.09	0.57	4.74	7.20	135
	West	6.07	0.75	4.20	7.28	180
Hospital	Overall	0.01	0.01	0.00	0.07	465
	East	0.01	0.00	0.00	0.07	150
	Middle	0.01	0.00	0.00	0.03	135
	West	0.00	0.00	0.00	0.03	180
Doctor	Overall	0.01	0.02	0.00	0.14	465
	East	0.02	0.01	0.00	0.14	150
	Middle	0.01	0.00	0.00	0.05	135
	West	0.00	0.03	0.00	0.03	180
Bed	Overall	0.03	0.04	0.00	0.29	465
	East	0.04	0.02	0.00	0.29	150
	Middle	0.02	0.00	0.00	0.03	135
	West	0.01	0.06	0.00	0.09	180
GDP	Overall	3.45	2.40	0.35	12.90	465
	East	5.19	2.91	0.82	12.90	150
	Middle	2.81	1.50	0.62	6.54	135
	West	2.48	1.59	0.35	7.19	180
Unemployment	Overall	0.04	0.01	0.01	0.07	465
	East	0.03	0.01	0.01	0.06	150
	Middle	0.08	0.01	0.02	0.07	135
	West	0.04	0.01	0.02	0.05	180
Inflation	Overall	2.68	1.86	−2.30	10.10	465
	East	2.49	1.74	−2.30	6.90	150
	Middle	2.68	1.78	−0.90	7.20	135
	West	2.84	1.99	−2.10	10.10	180
Density	Overall	4.36	6.45	0.02	40.43	465
	East	9.30	9.40	2.38	40.43	150
	Middle	3.04	1.45	0.82	6.07	135
	West	1.23	1.05	0.02	3.81	180
Birth	Overall	0.11	0.02	0.04	0.17	465
	East	0.10	0.02	0.04	0.17	150
	Middle	0.10	0.02	0.05	0.14	135
	West	0.12	0.02	0.07	0.17	180

The correlations matrix of this study is shown in Table 3. Table 3 shows that the independent variables of the number of doctors, hospitals, and beds are multicollinearity. Therefore, it is necessary to apply all independent variables independently to the model in order to examine more clearly the effect of each medical resource on mortality rate.

**Table 3:** Correlation statistics.

	**1**.	**2**.	**3**.	**4**.	**5**.	**6**.	**7**.	**8**.
1. Mortality	1							
2. Hospital	−0.30	1						
3. Bed	−0.20^***^	0.85^***^	1					
4. Doctor	−0.18^***^	0.78^***^	0.96^***^	1				
5. GDP	−0.23^***^	−0.08^*^	0.06	0.03	1			
6. Unemployment	0.26^***^	−0.11^**^	−0.18^***^	−0.11^**^	−0.40^***^	1		
7. Inflation	−0.02	−0.01	0.00	0.01	−0.13^**^	0.07	1	
8. Density	−0.16^***^	−0.16^***^	−0.04	−0.07	0.56^***^	−0.07	−0.05	1
9. Birth	0.08^*^	0.29^***^	0.26^***^	0.29^***^	−0.30^***^	−0.12^***^	0.03	−0.35^***^

*** *p < 0.01*,

** *p < 0.05*,

* *p < 0.1*.

In Table 4, the results show that the impact of hospitals, beds and doctors are statistically significant and negative. An increase in beds and hospitals significantly reduced mortality, while an increase in doctors had no significant effect on mortality. Thus, Hypothesis 1 can be accepted. The coefficient of hospitals, doctors, and beds are −12.81, −10.30, and −9.03, respectively. A one unit increase in hospitals, doctors and beds per residents will reduce the percentage of mortality by 12.81, 10.30, and 9.03, respectively. Increasing the number of hospitals nationwide would be most effective in reducing mortality. Increasing the number of beds would also have an effect in reducing mortality but increasing the number of doctors would not have a significant effect.

**Table 4:** China nationwide range's regression result.

**Dependent variables**	**Mortality**		
Hospital	−12.81^***^ [2.77] (1.11)		
Doctor		−10.30^***^ [9.28] (1.12)	
Bed			−9.03^**^ [2.04] (1.12)
GDP	−0.08^***^ [0.03] (1.94)	0.10^*^ [0.03] (1.97)	0.10 [0.03] (1.96)
Unemployment	20.29^***^ [13.18] (1.33)	19.40^***^ [12.24] (1.32)	12.48^**^ [11.13] (1.34)
Inflation	−0.01^*^ [0.00] (1.02)	−0.01 [0.00] (1.02)	−0.01 [0.00] (1.02)
Density	−0.14^***^ [0.03] (1.60)	−0.16^***^ [0.04] (1.59)	−0.16^***^ [0.03] (1.59)
Birth	2.10 [4.80] (1.32)	1.36 [4.91] (1.36)	1.32 [4.74] (1.33)
Constant	5.38^***^ [0.61]	5.68^***^ [0.68]	6.05^***^ [0.61]
Province FE	Yes	Yes	Yes
Year FE	Yes	Yes	Yes
Number of Observations	465	465	465
*F*-value	55.34^***^	2.23^***^	7.86^***^
Adj-R^2^	0.1427	0.1443	0.1863

* *p < 0.1*,

** *p < 0.05*,

*** *p < 0.01, the standard deviation is shown in square brackets, and variance inflation factor (VIF) are reported in parentheses*.

In Table 5, the impact of doctors is not significant and is negative in the eastern and middle regions. The impact of hospitals, doctors, and beds are statistically significant and negative in the western region. It indicates that the improvement of medical resources in the east and middle regions has no significant impact on mortality rate, while the improvement of medical resources in the western region has a significant impact on local mortality rate. Thus, Hypothesis 2 can be accepted. The coefficient of hospitals in the western regions is −16.51. A one unit increase in hospitals per residents will reduce the percentage of the mortality rate in the western regions by 16.51. The coefficient of doctors in the western regions is −8.74. A one unit increase in doctors per residents will reduce the percentage of mortality rate in the western regions by 8.74. The coefficient of beds in the west regions is −8.99. A one unit increase in beds per residents will reduce the percentage of mortality rate in the western regions by 8.99. This regression result shows that improving medical resource allocation in the western region is the most effective method to reducing the local mortality rate. Increasing medical resource allocation in the eastern region is more effective for reducing local mortality than in the middle region. Variance inflation factors (the range between 1.01 and 3.07) and Cook's distance statistics (the range between 0 and 0.02) from preliminary OLS regressions did not suggest issues of multicollinearity or influential observations.

**Table 5:** East, middle, west region in China regression result.

**Variables**	**East region mortality**			**Middle region mortality**			**West region mortality**		
Hospital	−14.47 [5.57] (1.12)			−12.41 [5.23] (2.10)			−16.51^***^ [3.53] (1.36)		
Doctor		−3.83 [3.48] (1.23)			−2.33 [1.17] (3.07)			−8.74^**^ [3.52] (1.74)	
Bed			−6.44 [6.17] (1.30)			−7.78 [6.36] (2.97)			−8.99^***^ [2.03] (1.63)
GDP	0.04 [0.03] (1.73)	0.04 [0.03] (1.72)	0.04 [0.03] (1.71)	0.21 [0.06] (1.83)	0.07 [2.54] (2.54)	0.16 [0.09] (1.74)	0.08 [0.05] (1.30)	0.11 [0.06] (1.37)	0.09 [0.05] (1.34)
Unemployment	−0.06 [11.44] (1.36)	12.86 [13.22] (1.45)	6.53 [14.37] (1.58)	12.53^***^ [9.32] (1.80)	6.70^***^ [13.26] (1.87)	7.59^**^ [11.77] (1.95)	52.91^***^ [13.80] (1.37)	52.53^***^ [10.60] (1.31)	36.20^***^ [14.91] (1.31)
Inflation	−0.01 [0.01] (1.02)	−0.01 [0.01] (1.01)	−0.01 [0.01] (1.01)	−0.01 [0.01] (1.09)	0.00 [0.01] (1.09)	−0.01 [0.01] (1.09)	−0.01 [0.00] (1.04)	−0.01 [0.00] (1.04)	−0.01^*^ [0.00] (1.04)
Density	−0.14^***^ [0.03] (2.19)	−0.10^**^ [0.03] (2.41)	−0.12^**^ [0.04] (2.37)	−0.35^***^ [0.70] (1.97)	−0.15^***^ [0.90] (3.01)	−0.17^***^ [0.95] (2.78)	0.29 [1.17] (1.28)	0.30 [1.13] (1.42)	0.32 [1.08] (1.39)
Birth	14.11^***^ [3.66] (1.57)	14.11^***^ [3.41] (1.50)	13.85 [3.76] (1.50)	8.43 [8.77] (2.05)	6.49 [9.05] (2.54)	7.17 [8.73] (2.50)	1.17 [7.89] (1.33)	0.94 [7.60] (1.56)	0.47 [7.11] (1.47)
Constant	5.34^***^ [0.59]	4.22^***^ [0.63]	4.80^***^ [0.69]	5.06^***^ [1.85]	5.58^***^ [2.61]	6.15^***^ [2.84]	3.48^**^ [7.89]	3.64^**^ [1.40]	4.40^**^ [1.64]
Province FE	Yes	Yes	Yes	Yes	Yes	Yes	Yes	Yes	yes
Year FE	Yes	Yes	Yes	Yes	Yes	Yes	Yes	Yes	Yes
Number of Observations	150	150	150	135	135	135	180	180	180
*F*-value	13.62^***^	18.16^***^	15.83^***^	5.37^***^	20.83^***^	4.86^***^	29.21^***^	9.87^***^	25.13^***^
Adj-R^2^	0.4146	0.4325	0.4139	0.1778	0.1942	0.1515	0.1658	0.1937	0.2379

* *p < 0.1*,

** *p < 0.05*,

*** *p < 0.01, the standard deviation is shown in square brackets, and variance inflation factor (VIF) are reported in parentheses*.

### Endogeneity Test

As noted above, the local GDP per capita, local unemployment rate controlled the mortality generated by socio-economic conditions and the difference in the availability of medical resources across regions. At the same time, considering the robustness of the study's conclusion, a robust standard error was used in the regression. In order to further alleviate the endogenous problem caused by omitted variable bias, the province and time fixed effect was used. Finally, for the reverse causality of medical resources on local mortality, the study refers to Chang and Kim (33) and uses population ratio as the instrumental variable. First, the population ratio of an area is significantly related to the level of local medical resources, while there is no significant correlation for mortality rate. Therefore, population ratio is used as the instrumental variable of the impact of medical resources on mortality in this study.

The results of 2SLS in Table 6, show that Hospitalf, Doctorf, and Bedf are fitted values of Hospital, Doctor, and Bed in 2SLS. The results show that the influence of Hospitalf Bedf, and Doctorf are statistically significant and negative. Thus, the regression result of the fitted value's coefficient is consistent with the regression result of independent variables of Hospital, Doctor, and Bed. The result of the study on controlling endogeneity resulting from reverse causality is basically valid.

**Table 6:** Endogeneity test by 2SLS.

**Dependent variables**	**Mortality**		
Hospitalf	−4.25^**^ [3.11] (1.02)		
Doctorf		−3.17^**^ [2.01] (1.09)	
Bedf			−3.27^***^ [3.21] (1.33)
GDP	−0.05^***^ [0.05] (1.04)	−0.06^***^ [0.03] (1.07)	−0.06^***^ [0.07] (1.09)
Unemployment	12.33^***^ [13.11] (1.14)	13.14^***^ [11.11] (1.14)	10.13^***^ [10.14] (1.15)
Inflation	−0.04^**^ [0.02] (1.21)	−0.07^**^ [0.04] (1.19)	−0.08^**^ [0.03] (1.24)
Density	−0.13^**^ [0.07] (1.13)	−0.13^**^ [0.08] (1.01)	−0.14^**^ [0.08] (1.28)
Birth	3.31 [6.31] (1.17)	4.21 [0.85] (1.33)	5.11 [1.47] (1.19)
Constant	6.77^***^ [0.51]	5.20^***^ [0.42]	5.11^***^ [0.55]
Province FE	Yes	Yes	Yes
Year FE	Yes	Yes	Yes
Number of observations	465	465	465
*F*-value	5.35^***^	6.03^***^	11.42^***^
Adj-R^2^	0.1647	0.1121	0.1721

** *p < 0.05*,

*** *p < 0.01, the standard deviation is shown in square brackets, and variance inflation factor (VIF) are reported in parentheses*.

## Conclusions and Policy Implications

There is no doubt that an increase in medical resources has improved the health of the population. With economic development in China, the distribution of medical resources is uneven. Although studies have been conducted on the impact of medical resources on residents' mortality rates, there is a lack of relevant studies on China. This study fills this gap through empirical research.

The empirical results of this study confirm that first, increasing medical resources nationwide can reduce the mortality rate, and increasing the number of hospitals doctors, and beds has a significant impact. Second, China has an unequal distribution of medical resources. The east and middle regions have more medical resources, while the west region has relatively less. Third, the improvement of medical resources in the west region results in a significant reduction of local mortality compared with that in the middle and east regions.

Based on the summary above, the following policy implications are proposed. First, China's nationwide medical resources are still inadequate, and improving medical resources throughout the country can reduce mortality rate. Second, due to the unequal distribution of medical resources in China, the Chinese government should implement more supportive policies for medical resources in the west region. Furthermore, we should also develop the west region, improve local per capita GDP, and reduce unemployment, so as to fundamentally improve the local economic level and reduce local mortality rate.

Some of the limitations of this empirical study are as follows. This study collects data from 31 provinces in China. However, in terms of the current situation in China, medical resources in each province are mostly concentrated in provincial capitals and large cities, while medical resources in small cities and rural areas are still insufficient. This phenomenon has resulted in residents of each province having to go to hospitals in nearby big cities. Due to limited data acquisition, this study was unable to collect relevant data in various small cities and villages in China. Therefore, this study can only carry out empirical research at the provincial level.

## Data Availability Statement

The datasets analyzed in this article are not publicly available. Requests to access the datasets should be directed to the corresponding author.

## Author Contributions

All authors designed and conducted the study, analyzed the data, wrote the daft, contributed to the interpretation of results, critically reviewed the draft, and approved the final manuscript.

### Conflict of Interest

The authors declare that the research was conducted in the absence of any commercial or financial relationships that could be construed as a potential conflict of interest.
